# Recombinant vaccines of a CD4^+^ T-cell epitope promote efficient control of *Paracoccidioides brasiliensis* burden by restraining primary organ infection

**DOI:** 10.1371/journal.pntd.0005927

**Published:** 2017-09-22

**Authors:** Rodrigo Assunção Holanda, Julián Esteban Muñoz, Lucas Santos Dias, Leandro Buffoni Roque Silva, Julliana Ribeiro Alves Santos, Sthefany Pagliari, Érica Leandro Marciano Vieira, Tatiane Alves Paixão, Carlos Pelleschi Taborda, Daniel Assis Santos, Oscar Bruña-Romero

**Affiliations:** 1 Departamento de Microbiologia, Universidade Federal de Minas Gerais, Minas Gerais, Brazil; 2 Laboratório de Biologia Parasitária, Universidade CEUMA, Maranhão, Brazil; 3 Departamento de Microbiologia, Universidade de São Paulo, São Paulo, Brazil; 4 Escuela de Medicina y Ciencias de la Salud, Universidad del Rosario, Bogotá, Colombia; 5 Laboratório de Microbiologia Ambiental, Universidade CEUMA, Maranhão, Brazil; 6 Departamento de Microbiologia, Imunologia e Parasitologia, Universidade Federal de Santa Catarina, Santa Catarina, Brazil; 7 Faculdade de Medicina, Universidade Federal de Minas Gerais, Minas Gerais, Brazil; 8 Departamento de Patologia Geral, Universidade Federal de Minas Gerais, Minas Gerais, Brazil; Saudi Ministry of Health, SAUDI ARABIA

## Abstract

Paracoccidioidomycosis (PCM) is an infectious disease endemic to South America, caused by the thermally dimorphic fungi *Paracoccidioides*. Currently, there is no effective human vaccine that can be used in prophylactic or therapeutic regimes. We tested the hypothesis that the immunogenicity of the immunodominant CD4^+^ T-cell epitope (P10) of *Paracoccidioides brasiliensis* gp43 antigen might be significantly enhanced by using a hepatitis B virus-derived particle (VLP) as an antigen carrier. This chimera was administered to mice as a (His)_6_-purified protein (rPbT) or a replication-deficient human type 5 adenoviral vector (rAdPbT) in an immunoprophylaxis assay. The highly virulent Pb18 yeast strain was used to challenge our vaccine candidates. Fungal challenge evoked robust P10-specific memory CD4^+^ T cells secreting protective Th-1 cytokines in most groups of immunized mice. Furthermore, the highest level of fungal burden control was achieved when rAdPbT was inoculated in a homologous prime-boost regimen, with 10-fold less CFU recovering than in non-vaccinated mice. Systemic Pb18 spreading was only prevented when rAdPbT was previously inoculated. In summary, we present here VLP/P10 formulations as vaccine candidates against PCM, some of which have demonstrated for the first time their ability to prevent progression of this pernicious fungal disease, which represents a significant social burden in developing countries.

## Author summary

Human paracoccidioidomycosis (PCM) represents a serious public health issue due to its disabling sequelae in working-age people and mortality rates (8^th^ among chronic infectious parasitic diseases in endemic countries and first among mycoses in Brazil). Although antifungal drugs have been widely used and provide clinical improvement for patients, the long duration of treatments (commonly from 6 to 12 months) has contributed to non-compliance and worsening of the disease in many cases. Induction of protective immune responses (either prophylactic or immune-therapeutic) remains the most cost-effective approach against most infectious agents. Members of our group have previously reported the protective properties of P10, a peptide contained in *Paracoccidioides brasiliensis* gp43 antigen, against PCM. However, the magnitude of the CD4^+^ T-cell responses elicited lacked the capacity of completely protecting experimental animals. We demonstrate here that immunogenic virus-like particles (VLP) carrying multiple copies of P10 peptide substantially improve P10-related cellular immunity in mice. This was particularly true when an adenoviral immunization vector expressed the chimeric VLP. Moreover, VLP/P10 formulations were capable of controlling the host fungal burden and prevented fungal systemic dissemination. The efficacy of diverse VLP/P10 formulations in murine PCM showed at least one promising candidate vaccine against human PCM.

## Introduction

Paracoccidioidomycosis (PCM) is a neglected dermal, mucosal, and respiratory disease endemic to South America, which is caused by the thermally dimorphic fungi *Paracoccidioides* spp. [1]. The acquisition of *Paracoccidioides* spp. occurs mainly by inhalation of air-borne fungal propagules [2]. In the lungs, morphological changes occur from mycelia to the yeast form, which leads to alveolar injuries, and lympho-haematogenous spreading may occur, depending upon host susceptibility. Inflammatory responses sustained by interferon gamma (IFNγ) and interleukin 2 (IL-2) are desirable to enhance microbial killing by macrophages and polymorphonuclear cells (PMN), whereas high levels of IL-4 and IL-10 are associated with the spread of fungus to other organs and worse prognosis [3,4].

Due to the limitations of antifungal therapies [3], immunogenic antigens, and in particular peptide P10 (amino acid sequence QTLIAIHTLAIRYAN), the immunodominant CD4^+^ T-cell epitope of the 43 kDa glycoprotein (gp43) from *P*. *brasiliensis*, were selected for vaccine purposes. P10 has the capacity to bind to major histocompatibility complex class II molecules from mice and humans [5,6]. Amongst P10-based vaccines, DNA vaccines containing P10 and IL-12 encoding sequences, and a P10 mixture with a TLR-5-engaging-molecule, i.e. flagellin, were the most effective formulations for controlling the fungal burden in the host [7,8]. Other vaccine strategies were also developed, such as recombinant proteins and radio-attenuated Pb18 yeast cells, which were also shown to be effective against *P*. *brasiliensis* infection, displaying a safe profile [9,10].

Chimeric virus-like particles (VLPs) are artificial structures in which relevant protective epitopes from pathogen antigens may be included. They behave as harmless but potent immune-stimulating scaffolds, representing a new strategy for vaccine development [11,12]. Hepatitis B virus core antigen (HBcAg) is considered an excellent vaccine carrier that self-assembles into VLPs [13]. Vaccines based on purified proteins or peptides, which are very efficient at inducing antibody responses, present some challenges at inducing T-cell responses, and frequently induce weak protection against intracellular infectious agents. However, proteins that self-assemble into VLPs can induce potent T-cell responses because they are rapidly engulfed and processed by macrophages and dendritic cells, the most efficient T-cell response stimulators [14]. Additionally, regulatory agencies already approved vaccines based on VLP for human use [11].

Live, replication-deficient recombinant viruses are also interesting platforms for delivering vaccines due to their safety profile [15–18] and the fact that some vectors deliver large amounts of foreign antigens directly into antigen-presenting cells, significantly inducing, in particular, cellular immunity [19,20]. Among recombinant viruses, adenoviral vectors are considered one of the most efficient platforms for vaccination, due to their capacity to elicit memory responses mediated by T lymphocytes. In recent years, and despite widely-spread concerns about pre-existing immunity blockade of heterologous antigen-specific responses, recombinant human type 5 adenovirus (HuAd5) vaccine candidates have shown high-efficiency in the induction of memory T cells and protection against infectious agents such as Ebola virus [15], *Trypanosoma cruzi* [19], *Leishmania infantum* [20], or *Mycobacterium tuberculosis* [21].

In this context, we developed two vaccine candidates based on the same VLP construct, both containing the main CD4^+^ T-cell-specific epitope from *P*. *brasiliensis* (P10) within the C-terminal portion of HBcAg (fused as alanine-flanking sequences to amino acid 179 of HBcAg). First candidate was a P10/(His)_6_-purified protein (rPbT), and the second was a recombinant P10/human type 5 adenoviral vector (rAdPbT). We show here the immunogenicity of these vaccine candidates when administered individually or combined in a prime-boost protocol, as well as the protection induced in mice against a highly pathogenic strain of fungus that can cause a severe disease in humans.

## Methods

### Recombinant vaccines

Initially, a synthetic, sequence verified, HASS-HBcAg-P10 cassette, contained in pUC57 plasmid, was unidirectionally cloned into the adenoviral shuttle vector pAdCMV-Link to form the pAdPbT plasmid (pAd-HASS-HBcAg-P10), using *Bam*HI/*Bgl*II and *Hind*III restriction sites. A recombinant adenovirus (rAdPbT) was generated by co-transfection of this pAdPbT shuttle vector together with a plasmid containing the human type 5 adenoviral E1/E3-deleted genome (pJM17) into HEK-293 cells [22]. rAdPbT-infected 293 cells were used for western blotting to verify expression of HBcAg/P10 chimera from the recombinant adenovirus sequences. A subsequent overnight CsCl gradient centrifugation allowed for the recovery of a high titer recombinant adenovirus [23].

In parallel, HBcAg-P10 and HBcAg-pp89 encoding sequences contained in pUC57 plasmids were separately cloned into the *Bam*HI and *Hind*III restriction sites of pET28a system (Novagen) to form pET28aPbT and pET28aCMV plasmids, respectively, then transformed into *E*. *coli* Rosetta (DE3) lineage (Novagen) for protein expression (rPbT and rCMV, respectively) under IPTG induction. Purification was performed using Ni^2+^ columns under denaturing conditions (Probond Purification Kit, Life Technologies), according to the manufacturer’s instructions. Each protein was dialyzed against refolding buffer (10 mM Tris; 0.5 mM DTT; 20% glycerol; pH 7.2) at 4°C for 16 h. SDS-PAGE and western blotting were performed to verify expression of recombinant HBcAgs.

### P10 peptide synthesis

P10 peptide sequence QTLIAIHTLAIRYAN was synthesized by F-moc solid-phase method by GenScript. The purity of the peptide was at least 95% as judged by HPLC.

### Ethics statement, animals and immunization protocol

**Fig 1:**
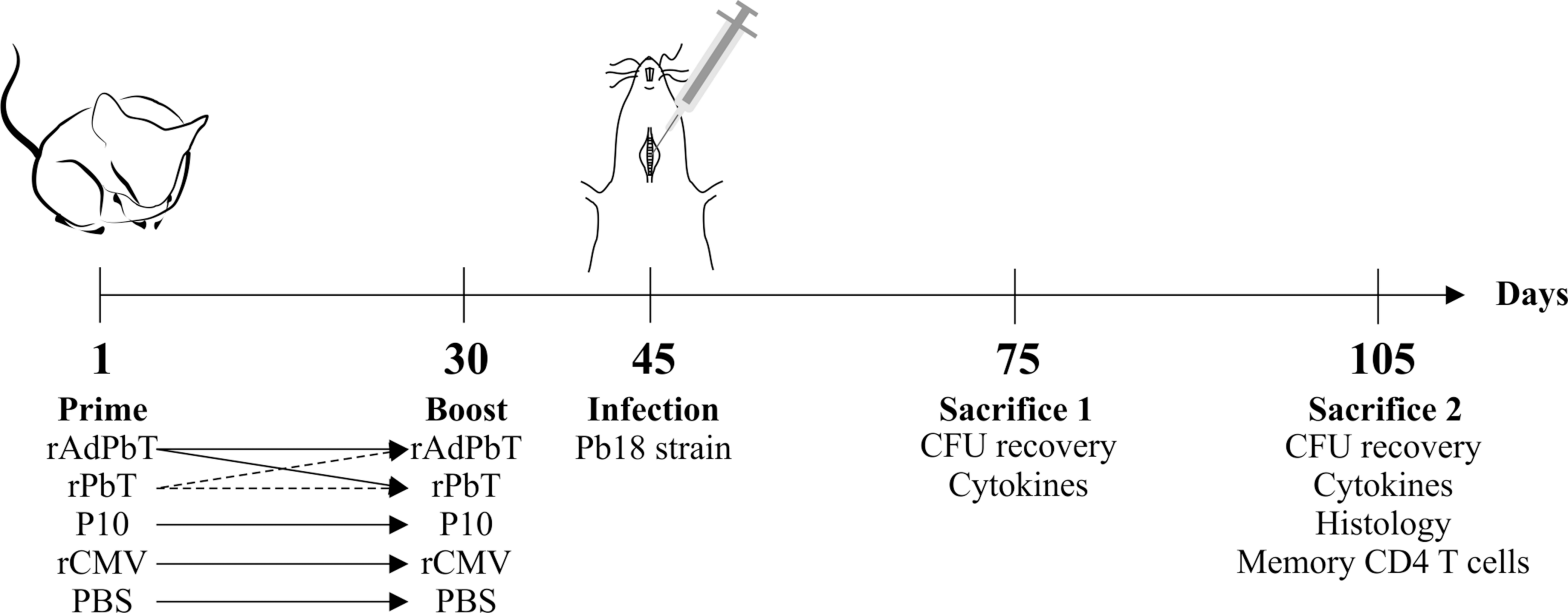
Scheme of murine immunization protocols used to test paracoccidioidomycosis prophylaxis. Mice were immunized subcutaneously with proteins or viruses as indicated (homologous or heterologous prime-boost protocols), and further challenged by intratracheal inoculation of Pb18 yeast cells. At the times indicated mice were sacrificed to evaluate tissue injuries, fungal burden, inflammatory responses, and humoral and/or cellular immune responses. rAdPbT is a recombinant adenovirus expressing a VLP/P10 chimera; rPbT is a purified recombinant VLP/P10 protein chimera; P10 is a synthetic peptide; rCMV is a purified recombinant VLP/pp89 protein chimera used as control; mock-immunized mice were challenged with Pb18 strain but previously non-immunized; PBS indicates phosphate buffered saline emulsified in adjuvant as control (non-immunized).

Animal experiments were carried out with strict adherence to the Ethics Commission on Animal Use (CEUA) from Federal University of Minas Gerais (UFMG), Brazil, (Protocol 206/11) and the Brazilian Federal Law 11,794 (October 8, 2008). Male BALB/c mice (6–8 weeks old) were purchased from UFMG´s central animal facilities, and housed in clean micro-isolator cages with food and water *ad libitum* for 12h light/dark cycles. Nine animals were assigned to each group. A detailed description of the prime-boost protocols used, vaccine candidates combinations and time frames (immunization intervals, challenge and sacrifice) are shown in Fig 1.

### Immunization procedures

Ten micrograms of recombinant proteins (rPbT or rCMV) or peptide (P10) were separately emulsified in Montanide ISA 720 adjuvant (Seppic, France) in a final volume of 100 μL. Recombinant adenovirus (rAdPbT) at 3×10^8^ plaque-forming units (PFU) was prepared in apyrogenic sterile phosphate buffered saline (PBS) supplemented with 1% normal mouse serum in a final volume of 100 μL.

Experimental groups were immunized with rAdPbT and rPbT in prime-boost homologous and heterologous protocols as shown in Fig 1. Control groups were immunized with P10 or rCMV or PBS emulsified in adjuvant in a homologous prime-boost protocol. All formulations were administrated subcutaneously in the tail base at the first and 30^th^ experimental days.

### Intratracheal infection

Mice were anesthetized with a solution containing ketamine hydrochloride (80 mg.kg^-1^) and xylazine (10 mg.kg^-1^) in sterile PBS, then inoculated intratracheally with 3×10^5^ viable yeast cells of virulent Pb18 strain [8]. Non-infected animals were inoculated with sterile PBS. Mice that were challenged with the Pb18 strain but previously non-immunized were called mock-immunized. Animals were monitored daily until euthanasia.

### Cytokine quantification

Inflammatory mediators such as IFNγ, TNF-α, IL-12, IL-10, and IL-4 were detected in 100 mg of lung homogenates using a BD Opteia ELISA Kit (BD Bioscience, USA), according the manufacturer’s instructions.

### Lymphoproliferation and immunophenotyping

Spleens were aseptically removed from mice and red blood cells were disrupted by osmotic lysis. Splenocytes (1.5×10^6^ viable cells) were labeled with 5 μM CFDA-SE (carboxyfluorescein succinimidyl ester, Sigma Aldrich, USA) [24], then incubated in RPMI 1640 medium supplemented with 10% of FBS and 40 mg/L of gentamycin for 80h at 37°C and 5% CO_2_. Positive (cell stimulation with ConA at 4 μg/mL) and negative (non-stimulated cells) controls of proliferation were performed using non-immunized and non-infected (NI) mice. Intratracheal Pb18 infection itself was the stimulus for evocation of memory CD4^+^ T-cell responses.

After 80h of restimulation, splenocytes were harvested and labeled with a panel of antibodies (anti-CD3-APC-Cy7, anti-CD4-PerCP-Cy5.5, anti-CD44-PE, and anti-CD62L-APC; BD Bioscience, USA). All samples were acquired in a BD FACSCanto II instrument (BD Bioscience, USA) and results were analyzed using FlowJo software v7.5 (TreeStar).

### Fungal burden measurement and histopathological analysis

Mice were euthanized for aseptic removal of the lungs, liver, and spleen. Each organ homogenate was plated onto Brain Heart Infusion agar plates (Difco, USA) supplemented with 4% FBS, 5% spent *P*. *brasiliensis* 192 strain culture supernatant, and 40 mg/L of gentamicin. Plates were incubated at 36°C for 20 days for yeast growth, and the colony-forming unit (CFU) counts were determined per gram of tissue. For evaluation of histopathological alterations, each organ was stained with hematoxylin-eosin (HE).

### Statistical analysis

All data were subjected to ANOVA (Tukey’s post hoc test) and Student’s *t-*test to analyze significant differences between groups. Results are shown as means ± standard deviation.

## Results

### Recombinant chimeras used as vaccine candidates

**Fig 2:**
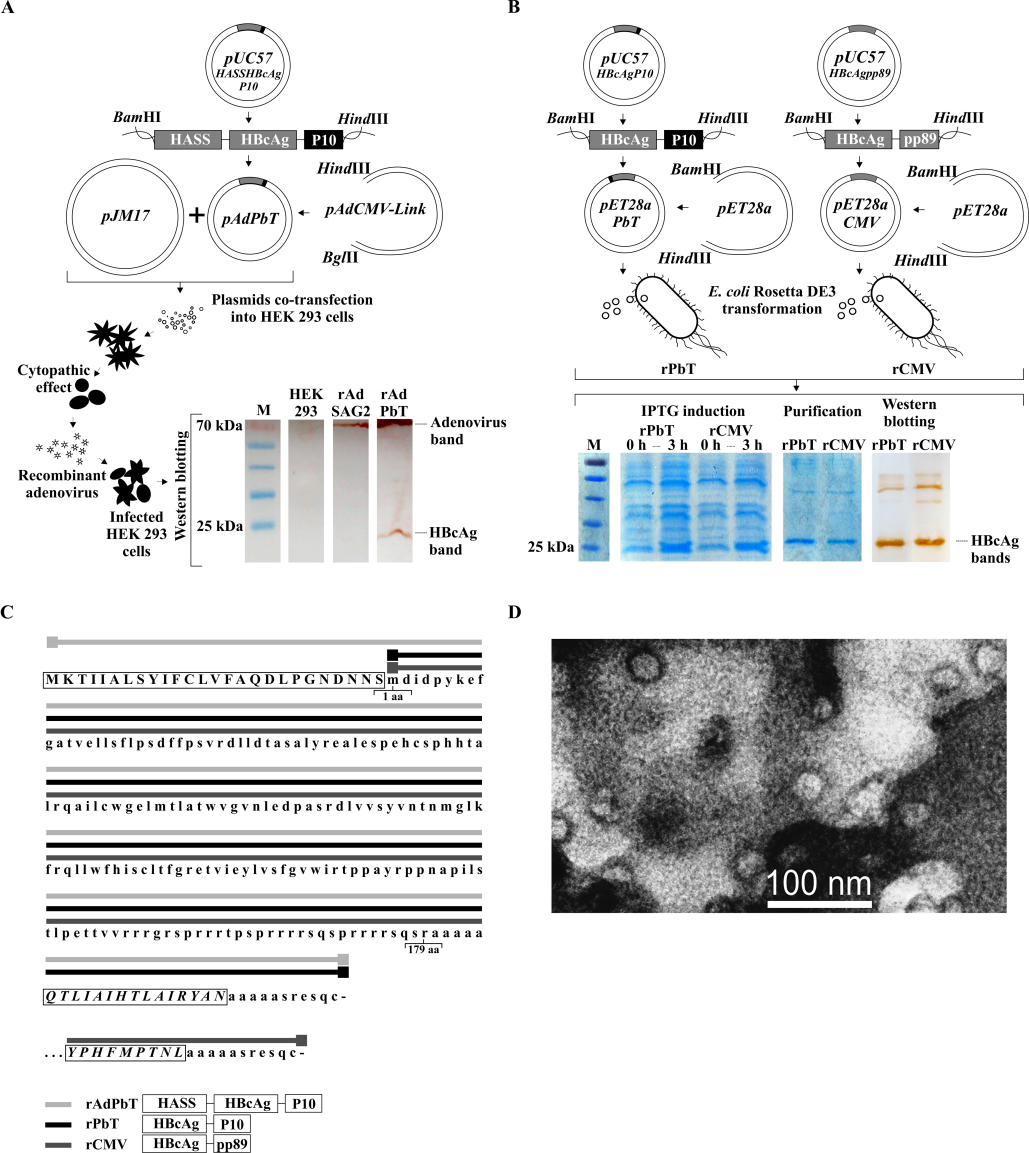
Recombinant VLP chimeras. (A) Cloning and co-transfection steps for generating rAdPbT adenoviral vector. Extracts of non-infected and adenovirus-infected HEK293 cells were incubated with dual anti-HBcAg/anti-adenoviral polyclonal sera. HBcAg and adenoviral proteins displayed 25 and 70 kDa bands, respectively. rAdSAG2 is a control recombinant adenovirus that encodes a surface antigen of *Toxoplasma gondii*. (B) Cloning, expression, purification and western blot reactivity tests of rPbT and rCMV (control) proteins from *E*. *coli* Rosetta DE3 bacteria. All HBcAg-derived bands display molecular weights of around 25 kDa. M = protein molecular weight ladder; 0h, 3h = times after IPTG induction. (C) Amino acid sequences of chimeric proteins, indicating the presence of signal peptide (HASS, uppercase letters within initial rectangle), hepatitis B virus core sequences (HBcAg, lowercase letters), *P*. *brasiliensis* epitope P10 (lower set of uppercase letters within a rectangle) or MCMV pp89 epitope (lowest set of uppercase letters within a rectangle). Amino acid residues comprising HASS-HBcAg-P10 (continuous light grey line following the letters), HBcAg-P10 (continuous black line following the letters) and HBcAg-pp89 (continuous dark grey line following the letters) are displayed. Amino-acids 1 and 179 of HBcAg are also indicated. (D) Transmission electron micrographs of the rPbT virus-like particles obtained from *E*. *coli* bacteria.

A genetic chimera constructed with sequences encoding hepatitis B virus core antigen (HBcAg) fused to those of the promiscuous and protective *P*. *brasiliensis* gp43 CD4^+^ T-cell epitope (P10) was used to generate two vaccine candidates, a recombinant adenovirus (rAdPbT) and a recombinant protein purified from *E*. *coli* (rPbT). An experimental control, containing a murine cytomegalovirus (MCMV) epitope from immediate early protein 1 (pp89) was also constructed and expressed in *E*. *coli*. An overview of the cloning steps and expression analyses are shown in Fig 2A and 2B. Amino acid sequences of HBcAg-chimeric VLP constructs are shown in Fig 2C. VLPs assembly of our vaccine candidate was demonstrated by transmission-electron microscopy, and shown in Fig 2D.

### Cytokine profiles

Inflammatory responses induced by experimental PCM were quantified in lung homogenates at the 75^th^ and 105^th^ days.

**Fig 3:**
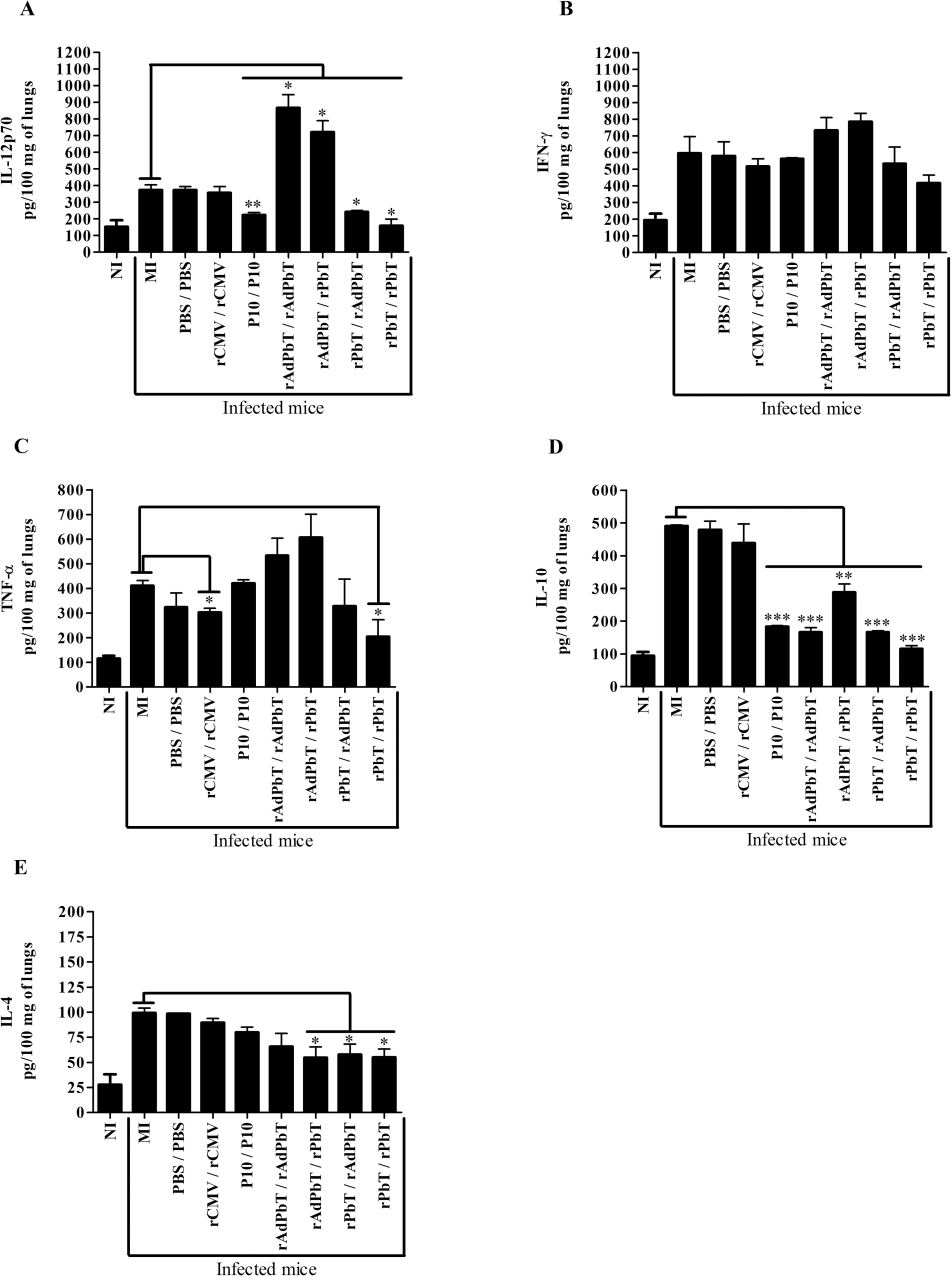
Cytokine quantification in lungs at the 75^th^ day. IL12p70 (A), IFN-γ (B), TNF-α (C), IL-10 (D) and IL-4 (E) were quantified in 100 mg of lung homogenates from the different groups of mice. Mock-immunized (MI), PBS/PBS, rCMV/rCMV, P10/P10, rAdPbT/rAdPbT, rAdPbT/rPbT, rPbT/rAdPbT and rPbT/rPbT mice were all intratracheally inoculated with virulent Pb18 yeast cells, while non-infected mice (NI) were intratracheally inoculated with sterile PBS. Data represent the mean of two independent experiments (three animals per experiment). ***(*p*<0,001), **(*p*<0,01) and *(*p*<0,05) were considered to be significant.

At the 75^th^ day, lungs of mice previously immunized with rAdPbT/rAdPbT and rAdPbT/rPbT protocols secreted the highest levels of IL-12 compared with mock-immunized (MI) or non-immunized (NI) mice, while other P10-based formulations secreted lower levels of this cytokine (Fig 3A). IFN-γ or TNF-α secretion was not statistically different after challenge among infected mice (Fig 3B and 3C) although differences were highly significant when compared to non-infected animals, suggesting that Pb18 infection strongly boosts production of these cytokines in all animals masking any previous differences due to vaccination. Interleukin-10 was less secreted in lungs of mice immunized with P10-based formulations (Fig 3D), while IL-4 was marginally less secreted only in those groups that received any of the P10/VLP constructs, i.e. rAdPbT/rPbT, rPbT/rAdPbT and rPbT/rPbT, when compared to mock-immunized mice (Fig 3E).

**Fig 4:**
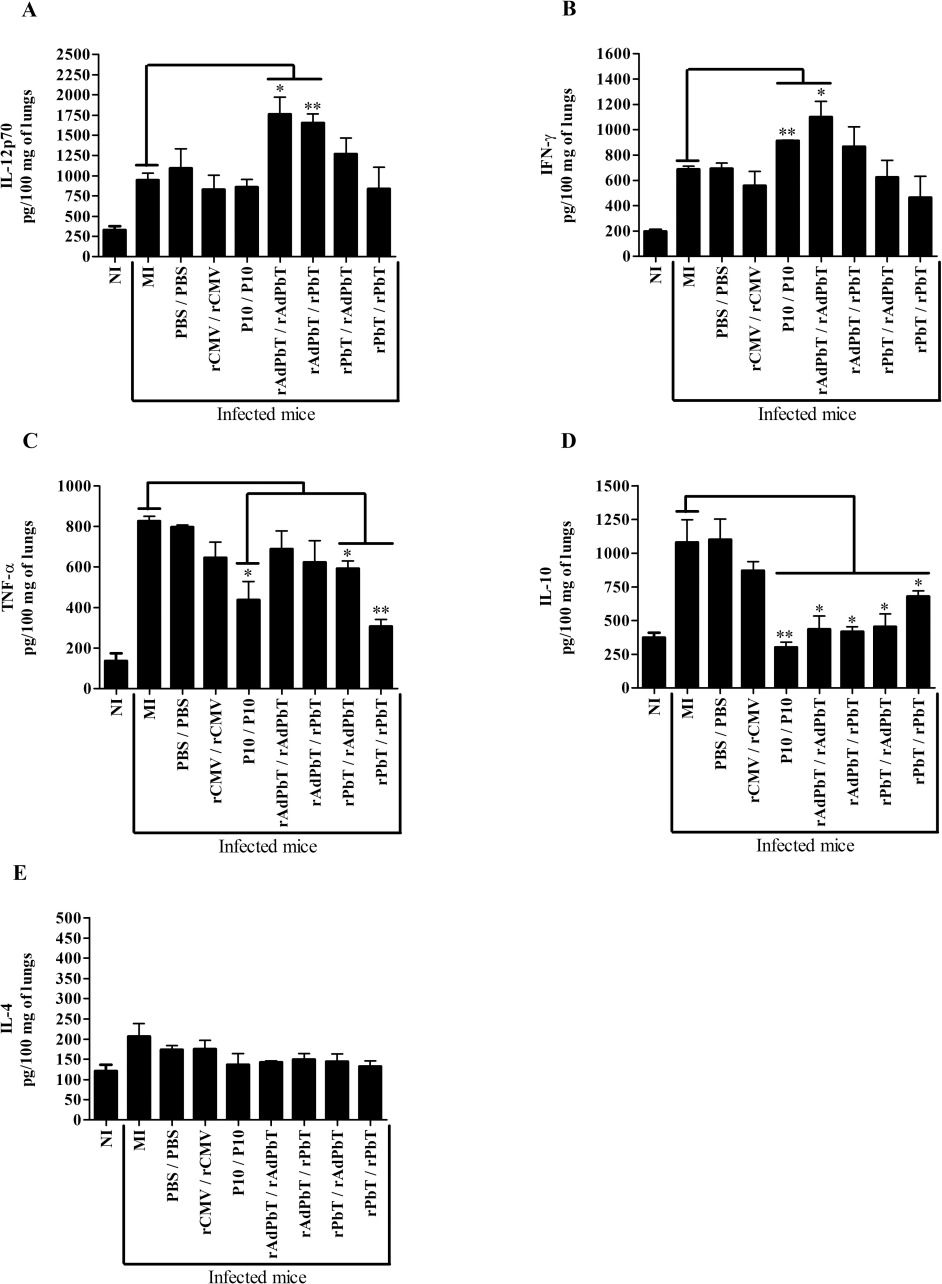
Cytokine quantification in lungs at the 105^th^ day. IL12p70 (A), IFN-γ (B), TNF-α (C), IL-10 (D) and IL-4 (E) were quantified from 100 mg of lung homogenate. Mock-immunized (MI), PBS/PBS, rCMV/rCMV, P10/P10, rAdPbT/rAdPbT, rAdPbT/rPbT, rPbT/rAdPbT and rPbT/rPbT were groups intratracheally inoculated with virulent Pb18 yeast cells, while non-infected mice (NI) were intratracheally inoculated with sterile PBS. Data represent the mean of two independent experiments (three animals per experiment). ***(*p*<0,001), **(*p*<0,01) and *(*p*<0,05) were considered to be significant.

As had been observed at day 75, lungs of rAdPbT/rAdPbT- and rAdPbT/rPbT-immunized mice also secreted the highest levels of IL-12 compared to mock-immunized mice at day 105 (Fig 4A), while all other mice immunized with P10-based formulations displayed levels of this cytokine that were not statistically different (a borderline significance was observed in some cases for group rPbT/rAdPbT). Regarding IFNγ, the lungs of rAdPbT/rAdPbT secreted more cytokine than mock-immunized mice (Fig 4B). As for TNF-α secretion, no group secreted this inflammatory cytokine at levels above those of mock-immunized mice (Fig 4C). As previously noted, interleukin-10 was significantly less secreted in lungs of mice previously immunized with P10-based formulations in relation to mock-immunized mice (Fig 4D). Finally, interleukin-4 was not statistical different between all infected mice (Fig 4E).

### Proliferative memory CD4^+^ T cells recalled by intratracheal Pb18 infection

**Fig 5:**
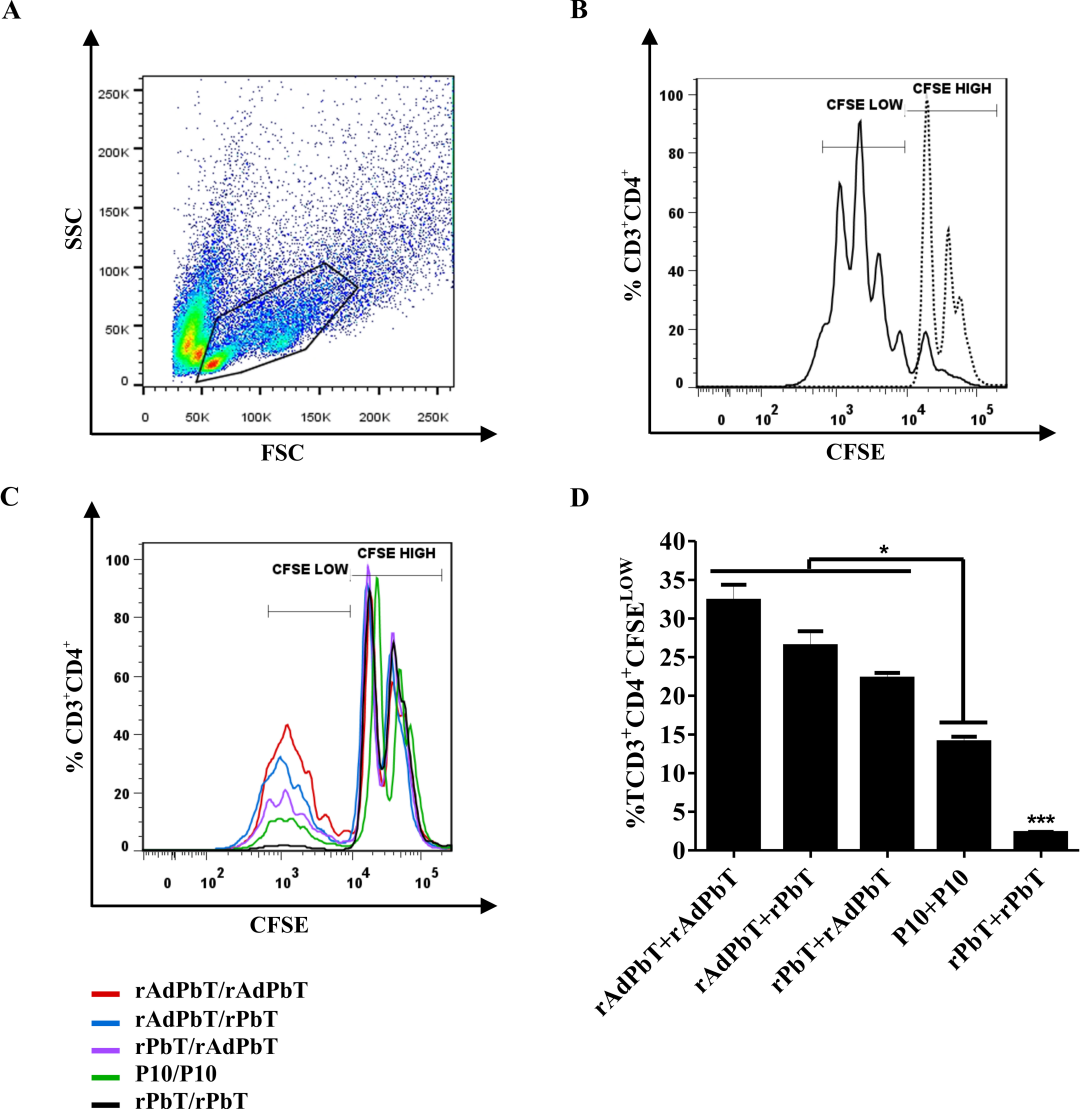
Specific proliferation of CFSE-labeled CD4^+^ T lymphocytes. Cellular immunity recalled by Pb18 infection in immunized (rAdPbT/rAdPbT, rAdPbT/rPbT, rPbT/rAdPbT, rPbT/rPbT, P10/P10, rCMV/rCMV and PBS/PBS) and mock-immunized (MI) mice at the 105^th^ experimentation day (75 days after the last immunization). (A) Lymphocyte population was delimited based on its size (FSC) and granularity (SSC) and CD4^+^ T lymphocytes were searched for by anti-CD3 and anti-CD4 surface marking. (B) Controls of non-stimulated proliferative CD4^+^ T cells were monitored in CD3^+^CD4^+^CFSE^HIGH^ histogram peaks (non-specific proliferation; dotted line) and ConA-stimulated CD4^+^ T cells were monitored in CD3^+^CD4^+^CFSE^LOW^ histogram peaks (continuous line) using splenocytes from non-infected and non-immunized mice. Proliferative CD4^+^ T cells of rAdPbT/rAdPbT-immunized mice (red line), rAdPbT/rPbT (blue line), rPbT/rAdPbT (purple line), P10/P10 (green line) and rPbT/rPbT-immunized mice (black line) recalled by intratracheal Pb18-challenge are shown as overlaid histograms (C) and the corresponding percentage of cells that experimented proliferation (D). Data represent the mean of two independent experiments (three animals per experiment). ***(*p*<0,001), **(*p*<0,01) and *(*p*<0,05) were considered to be significant.

Proliferative CD4^+^ T cells (Fig 5A) were monitored in CFSE histograms, where CD3^+^CD4^+^CFSE^HIGH^ dotted lines histogram represents non-specific proliferation and CD3^+^CD4^+^CFSE^LOW^ continuous line histogram represents specific proliferation after Pb18 infection, as shown in Fig 5B.

As expected, CD4^+^ T lymphocytes of all infected mice previously immunized with P10-based formulations displayed proliferative responses. The highest proliferative response was displayed in the rAdPbT/rAdPbT group (red line; ~32%) followed by the rAdPbT/rPbT (blue line; ~26%), rPbT/rAdPbT (purple line; ~22%), P10/P10 (green line; ~15%), and rPbT/rPbT groups (black line; ~2.5%), according to Fig 5C and 5D, respectively. CD4^+^ T cells of rCMV/rCMV, PBS/PBS, and mock-immunized mice were unable to proliferate in CFSE^LOW^ region (<1%).

**Fig 6:**
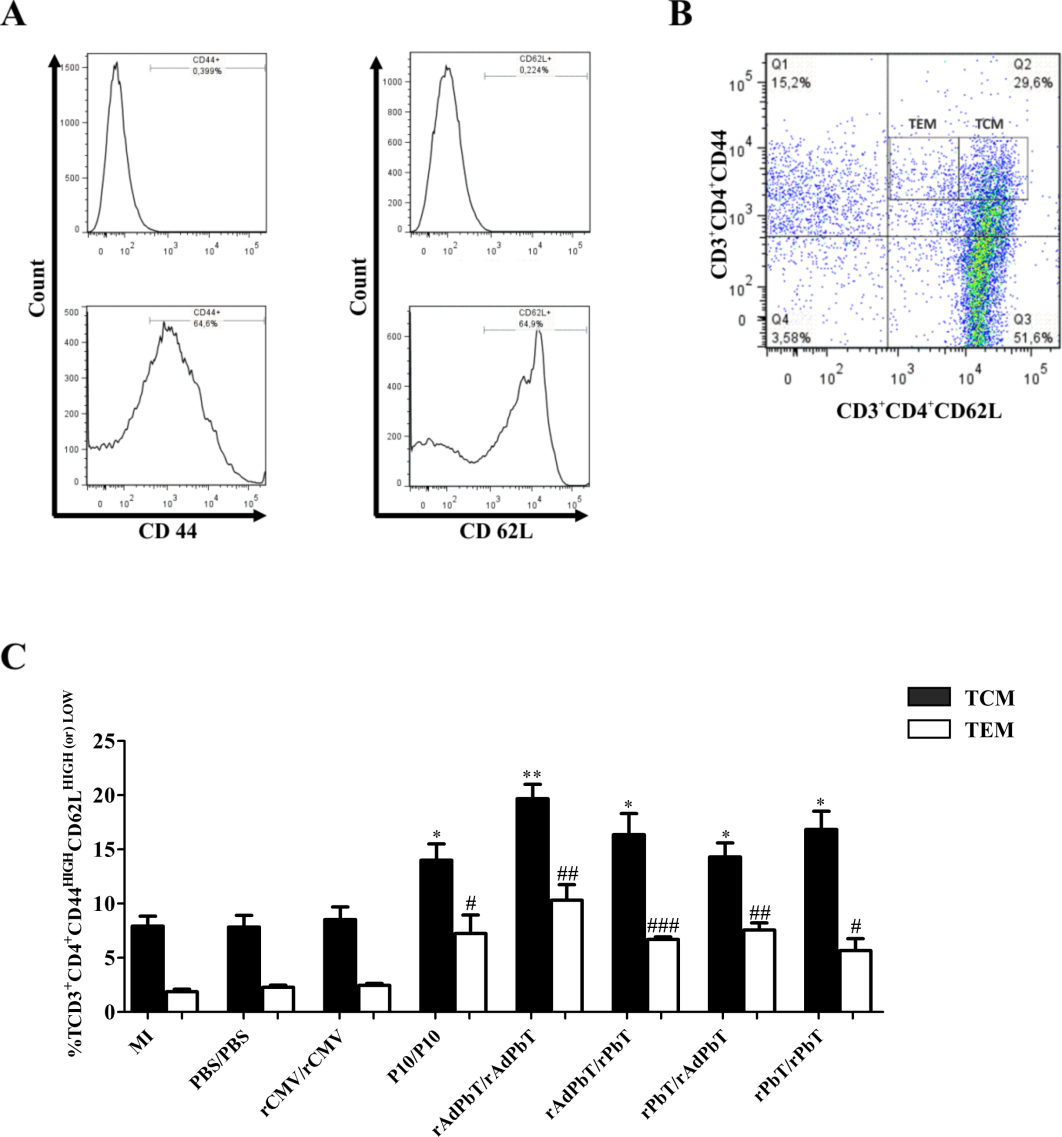
Memory CD4^+^ T lymphocytes recalled by *P*. *brasiliensis* infection. Central (TCM) and effector (TEM) phenotyping were evaluated at the 105^th^ experimentation day (75 days after the last immunization) in CD4^+^ T lymphocytes region (CD3^+^CD4^+^) using anti-CD44 and anti-CD62L surface marking, according to the staining controls for negative and positive populations (A). TCM and TEM phenotypes were evidenced in CD3^+^CD4^+^CD44^HIGH^CD62L^HIGH^ (filled bars) and in CD3^+^CD4^+^CD44^HIGH^CD62L^LOW^ (open bars) regions, respectively (B), as well as the percentages of these cells were calculated (C). Mock-immunized (MI), PBS/PBS, rCMV/rCMV, P10/P10, rAdPbT/rAdPbT, rAdPbT/rPbT, rPbT/rAdPbT and rPbT/rPbT were groups intratracheally inoculated by virulent Pb18 yeast. Data represent the mean of two independent experiments (three animals per experiment). *** or ### (*p*<0,001), ** or ## (*p*<0,01) and * or # (*p*<0,05) were considered to be significant.

Next, we investigated memory CD4^+^ T-cell phenotypes that were evoked after Pb18-challenge and the influence of immunoprophylaxis previously performed, based on circulating cells expressing activation (CD44) and homing (CD62L) cell-surface molecules (Fig 6A and 6B).

The central memory CD4^+^ T-lymphocyte (TCM) phenotype (CD3^+^CD4^+^CD44^HIGH^CD62L^HIGH^) was predominantly displayed in mice previously immunized with P10-based formulations. Amongst them, the rAdPbT/rAdPbT group displayed the most expressive TCM phenotype (~20%), followed by the rAdPbT/rPbT and rPbT/rPbT (~16%), then the rPbT/rAdPbT and P10/P10 (~14%) groups. The TCM phenotype of mice vaccinated with P10-based formulations was percentually more expressive than that of rCMV/rCMV, PBS/PBS, and mock-immunized mice (~8%), as shown in Fig 6C (black bars). We also verified the effector memory (TEM) phenotype (CD3^+^CD4^+^CD44^HIGH^CD62L^LOW^), which was more expressed in the rAdPbT/rAdPbT group (~10%). Unexpectedly, the rAdPbT/rPbT, rPbT/rAdPbT, rPbT/rPbT, and P10/P10 groups displayed similar responses of effector T cells (~ 6%), which were higher than those of the rCMV/rCMV, PBS/PBS, and mock-immunized groups (~2.5%), as shown in Fig 6C (white bars).

### Prophylactic vaccination with P10-based formulations extensively reduces the fungal burden in mice

To verify the protection induced by P10-based vaccines against *P*. *brasiliensis* infection, the fungal burden was recovered from the lungs, liver, and spleen at the 75^th^ and 105^th^ days.

**Fig 7:**
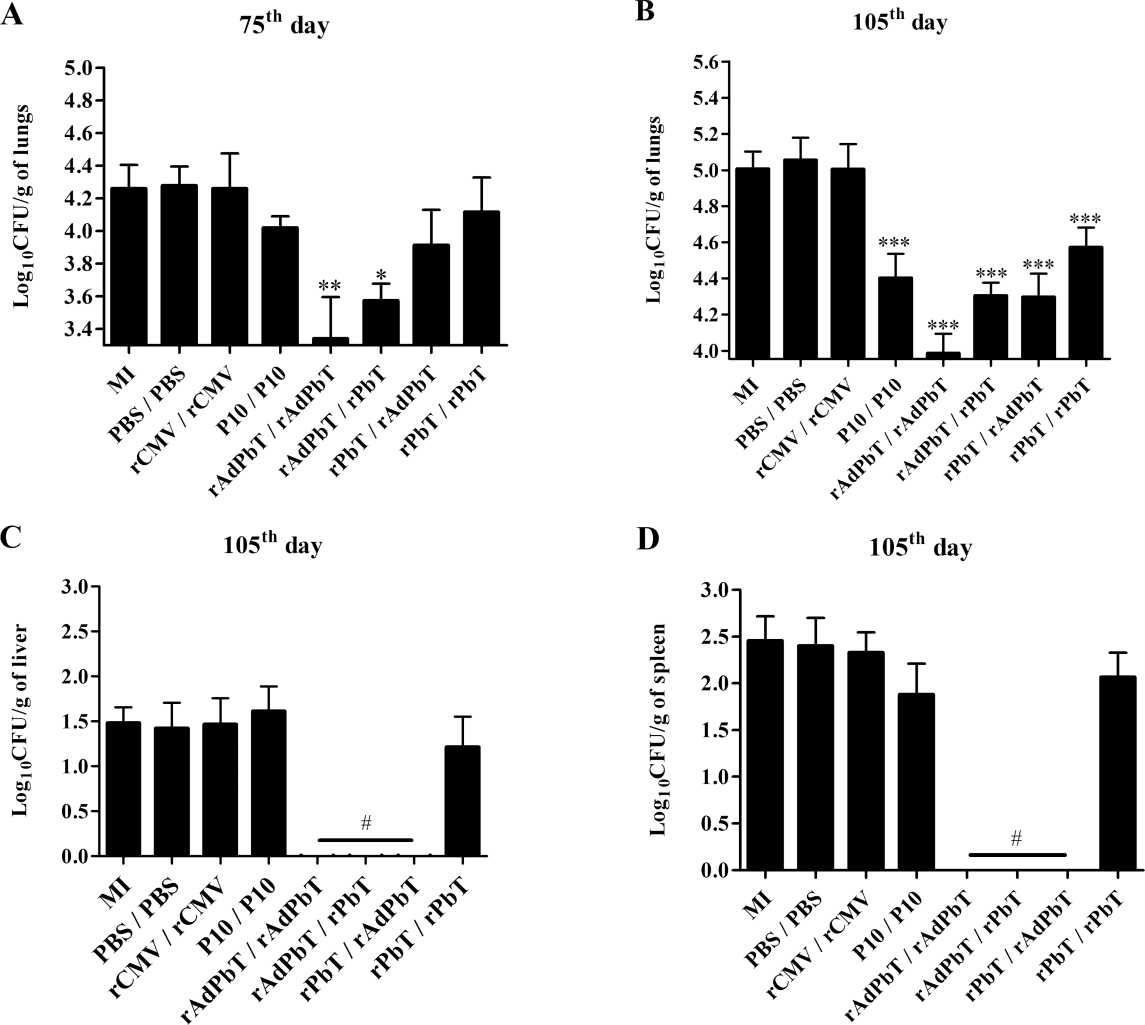
Fungal burden measurement. Plots representing the numbers of colony-forming units detected per gram of tissue at the 75^th^ and the 105^th^ days, as indicated, in different organs. (A and B) lungs; (C) livers; (D) spleens. All groups were intratracheally inoculated with virulent Pb18 yeast. Data represent the mean of two independent experiments (six animals per experiment). ***(*p*<0,001), **(*p*<0,01), *(*p*<0,05) and # (*p*<0,0001) were considered to be significant.

Unexpectedly, rPbT/rAdPbT, rPbT/rPbT, and P10/P10 were unable to reduce the fungal burden in the lungs at the 75^th^ day. On the other hand, mice immunized with a homologous adenovirus prime-boost regimen (rAdPbT/rAdPbT) showed a pronounced reduction in the fungal burden in the lungs (8-fold less than mock-infected mice), and a heterologous protocol initiated with adenovirus inoculation (rAdPbT/rPbT) was able to reduce the fungal burden (4-fold less than mock-immunized mice), as shown in Fig 7A. Mice immunized with control protein (rCMV/rCMV) or with adjuvant in PBS (PBS/PBS) displayed a similar fungal burden to that of mock-immunized mice. Colony-forming units were not recovered in the liver or spleen at the 75^th^ day.

Fortunately, all P10-based formulations (rAdPbT/rPbT, rPbT/rAdPbT, rPbT/rPbT, and P10/P10) were able to control fungal proliferation in the lungs compared with the mock-immunized group at the 105^th^ day (Fig 7B). As previously noted, rAdPbT/rAdPbT was again the most effective protocol to control the fungal burden in the lungs (10-fold less than mock-immunized mice), followed by rAdPbT/rPbT and rPbT/rAdPbT (5-fold less than mock-immunized mice), then P10/P10 and rPbT/rPbT-immunized mice (2-fold less than mock-immunized mice). Unexpectedly, the fungal burden in the lungs of all mice was higher at the 105^th^ day than those observed at the 75th day, highlighting the efficacy of our virulent Pb18 strain in the immunoprophylaxis assay. However, mice immunized with rAdPbT in the prime and/or boost regimen were able to prevent the fungus spreading from the lungs to other organs (Fig 7C and 7D).

### Histopathology

**Fig 8:**
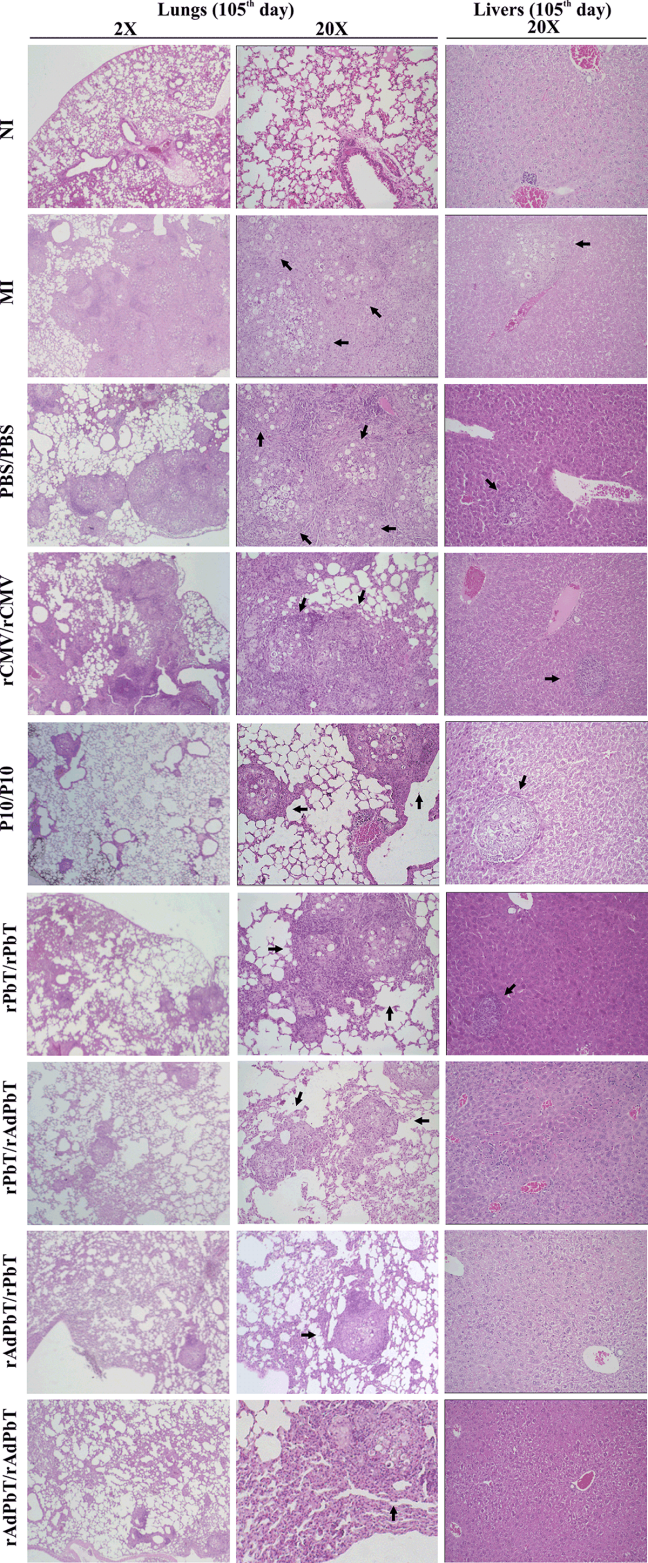
Histopathology. Lungs and livers were stained with Hematoxilin-Eosin at day 105 (2-20X magnification). Organs of normal uninfected mice intratracheally inoculated with sterile PBS (NI) and of those challenged with virulent Pb18 yeast after immunization (MI, PBS/PBS, rCMV/rCMV, P10/P10, rAdPbT/rAdPbT, rAdPbT/rPbT, rPbT/rAdPbT and rPbT/rPbT) were evaluated. Arrows indicate the presence of granulomas containing yeast cells.

Tissue injuries caused by virulent Pb18 infection were observed in organs sectioned at the 105^th^ day (Fig 8). Yeast cells were found associated with alveolar injuries in an independent manner in immunized and non-immunized mice. Furthermore, infiltration of foam macrophages and neutrophils were found in the bronchial lumen, and alveolar thickening was also observed.

Granulomas containing yeast cells were observed in the lung parenchyma of all infected mice, as expected because is this organ which receives the challenge microorganisms in the first place. On the other hand, it is of enormous interest the fact that there were no yeast cells in liver or spleen sections of any animal that received at least one dose of rAdPbT, the adenovirus-immunized groups of mice, whereas some hepatic granulomas containing yeast cells and neutrophil infiltration and even some free individual yeast cells were found in the rPbT/rPbT, P10/P10, rCMV/rCMV, PBS/PBS, and mock-immunized groups.

## Discussion

The main challenge in vaccination is how to induce a long-lasting protective memory against microbes and their antigens. Selection of candidate vaccine antigens, vectors, dosage, and vaccination protocols are the keys to improving the immunogenicity and efficacy of the formulation [25,26]. In paracoccidioidomycosis, the stimulation of Th-1 cells is desirable to activate macrophages associated with the clearance of microbes in the intracellular environment [4,27]. Recombinant VLP/P10 formulations were made in an attempt to improve P10 immunogenicity by presenting it to the immune system and to enhance microbial killing mediated by cellular immunity.

P10/HBcAg chimeras were expressed either in cells infected with a recombinant adenoviral vector (rAdPbT) or as recombinant *E*. *coli* His(6)-purified proteins (rCMV and rPbT) and self-assembled into VLPs according to our transmission electron microscopy data.

Cytokine profiles of the mock-immunized, PBS/PBS and rCMV/rCMV groups suggested the occurrence of tissue injury and modulation of the inflammatory response by a Th2-biased immune response. These data corroborate the poor CD4^+^ T lymphocyte response evoked by intratracheal Pb18 challenge in these groups.

In a PCM immunoprophylaxis study, a DNA vaccine based on HSP65 from *Mycobacterium leprae* induced a protective immune response against *P*. *brasiliensis* infection via high-level secretion of cytokines IFNγ and IL-12 [28]. In our study, the recombinant VLP protein carrying a specific CD8 T-cell epitope from murine cytomegalovirus (rCMV) did not cross-protect mice against *P*. *brasiliensis* proliferation, demonstrating the specificity of the CD4^+^ T cell response for protection, and allowing the use of that chimera for actual evaluation of P10-based vaccine efficacy.

Braun and colleagues [14] demonstrated that a VLP vaccine could elicit both humoral and cellular immune responses, as did Almeida and colleagues with *Plasmodium* CSP antigen expressed on the VLP surface [13]. The efficacy of VLP vaccines was also shown in clinical trials against human papilloma virus (HPV) where volunteers were entirely protected against a new infection by some viral serotypes [29]. In our study, VLP/P10 vaccines could efficiently induce protective memory P10-specific CD4^+^ T lymphocytes.

Immunization of mice with synthetic P10 peptide or purified VLP/P10 protein led to a delayed reduction of the fungal burden in the lungs that could be related to the decreased spread of Pb18 in the host. Concerning synthetic P10 vaccination, a significant protective response against *P*. *brasiliensis* infection was expected, in view of previous results obtained by members of our group [8]. Thus, Taborda and colleagues [30] had shown the protective immune responses induced against murine PCM by P10 peptide, either by using different adjuvants (Freund´s complete and incomplete or Flagellin), doses (1–20 μg), vaccination protocols (prime-boost with repetitions) and routes of inoculation (intranasal, subcutaneous, and intraperitoneal) [8,30,31]. However, purified VLP/P10 protein given by a homologous prime-boost protocol induced a low proliferative response of CD4^+^ T lymphocytes evoked by P18 infection and a limited, though significant, control of the fungal burden in mice.

In the present study, synthetic P10 peptide and purified recombinant proteins were emulsified in Montanide ISA 720 adjuvant, composed of natural metabolizable oil and a highly refined emulsifier from the mannide monooleate family [32], which is allowed for vaccine trial [33] and is able to induce the switch towards Th1 responses [33,34]. There is no need to add microorganisms or microbial products to this formulation, as opposed to Freund’s complete emulsion [8,30,31] and some Alum preparations [35,36].

In standardization experiments (S1 Fig) the subcutaneous route was the most immunogenic when considering both the protein chimera and the adenovirus vector. This had been also previously shown for rPbT [13] as well as for the adenovirus vector (in all of our previous publications on this subject since 1986). Regarding immunization with P10 peptide in adjuvant, we could not try to improve immunogenicity by increasing the frequency of inoculations, because maintaining the same time intervals among immunization would impede subsequent yeast challenge with Pb18 (it has to be done in adolescent to young adults mice) [7–10, 28, 31, 35,36], while if shortening the inoculation intervals, immunogenicity would have decreased due to lymphocyte Activation-Induced Cell Death (AICD) [37].

Recombinant adenovirus inoculation was substantially more immunogenic than purified recombinant proteins or synthetic P10 peptide, even when the viral vector was used in a homologous prime-boost protocol, something already observed in previous studies [21], demonstrating that preexisting host immunity against the vector was not enough to affect its immunogenicity or its capacity to delivery foreign antigens directly into antigen presenting cells. The highest secretion of proinflammatory cytokines from memory CD4^+^ T cells (central and effector phenotypes) was achieved when mice were primed with recombinant adenovirus in prime-boost regimens. This immune response profile is desirable for the clearance of infectious agents in the intracellular environment [4]. Of interest too is the fact that all groups of mice immunized with a P10-derived construct secreted significantly less IL-10 cytokine in the lungs than those mice mock-immunized, suggesting the induction of a focused and favorable Th1 immune response, with little regulation, in the main organ were lymphocyte effector functions should be displayed. More importantly, all mice immunized at some point with the recombinant adenovirus were protected against Pb18 systemic dissemination.

It has been shown that attenuated Pb18 yeast formulation, one of the most promising vaccine candidates against PCM, can evoke Th-1/Th-2 responses to control the host fungal burden [10]. In addition, recombinant Pb27 and Pb40 proteins based on Pb18-fractionated antigens have been shown to play an important role in defense against *P*. *brasiliensis* infection [35,36]. However, the mechanisms by which these formulations elicit a protective immune response are unclear. Immunophenotyping assays should be used to clarify the immunological memory elicited by whole and fractionated Pb18 antigens, which culminates in microbial killing.

Among live vaccines, recombinant adenoviral vectors, in particular, human type 5 adenovirus (Hu5Ad) are considered the most powerful activators of T lymphocytes [38,39]. In experimental trypanosomiasis, HuAd5-based vaccines constructed by us strongly elicited memory T cells that led to a pronounced reduction in parasitemia, an augmentation of mouse survival, and a regression of chronic cardiomyopathy [19]. In *M*. *tuberculosis*, a recombinant HuAd5 vaccine elicited a robust immune response of T lymphocytes when the vector was inoculated alone or as a booster regimen in volunteers previously immunized with BCG formulation, including individuals already pre-sensitized with Hu5Ad. The vaccine was well tolerated, effective, and safe for human use [21]. In this context, we believe that our replication-deficient rAdPbT vector, also built from the Hu5Ad genome, may be effective in patients suffering from PCM, as well as for immunization of individuals whose occupation may predispose them to *P*. *brasiliensis* infection. The main advantages of recombinant adenoviruses in relation to subunit vaccines are: i) they are powerful innate immune activators of Toll-like receptor and MyD88 pathways, and there is no need to use adjuvant with them, ii) they have the capacity to deliver high amounts of foreign antigens to the host, iii) they mimic intracellular infection by microbes, improving antigen presentation to the immune system iv) they are activators of T lymphocytes in both innate and adaptive immune responses. Furthermore, recombinant adenoviral vectors have some advantages over other live vaccine candidates: i) non-replicative ability in the host; ii) non-integrative ability in the host genome; iii) very low oncogenic potential; iv) facility of scaling up production; and v) target antigen expression [21,40,41].

In contrast with other mouse strains in which *P*. *brasiliensis* either leads to an acute and fatal outcome, e.g. in B10.A mice, or to a chronic and regressive self-healing disease, i.e. the disease observed in A/Sn or A/J mice [42,43], in the BALB/c strain of mice used in our study, Pb18 displayed an intermediate behavior [44], more similar to the human PCM for which we used those mice as model. Thus, albeit fungal burden didn’t lead mice to death until the day of sacrifice, something that would require immunosuppression with dexamethasone [31] thus invalidating any of our current observations and conclusions, lymphohaematogenous dissemination, related to clinical worsening [45], was significantly suppressed by the use of some of our vaccine candidates.

Regarding tissue injuries, granulomas surrounding yeast cells and cellular infiltrate were found in the lungs of all mice with PCM as expected. However, the containment of yeast cells inside granulomas in lung parenchyma seemed to be more efficient in mice that received at least one adenovirus dosage in the prime-boost protocol, which was enough to prevent the fungus from spreading to other organs. These results corroborate with the CFU numbers recovered from organs of those mice that displayed resistance against PCM [46–47].

Eventually, our recombinant vaccines may be used in a PCM therapeutic regimen [36] or, in a challenging approach, as tools in cancer therapy [48–51] due to the parallel antitumor properties that have been shown for P10 [52].

In conclusion, some of our recombinant VLP/P10 formulations, inoculated in homologous and heterologous prime-boost protocols, elicited a strong, long-lasting cell-mediated immune response that led to intense control of local infection and prevented the systemic spread of the fungus in the host.

## Supporting information

S1 Fig Routes of VLP/P10 vaccines administration. Detection of IgG anti-HBcAg in sera of mice immunized with rPbT and rAdPbT collected 15 days after immunization. rPbT was administered subcutaneously at 1, 5, 10 and 25 μg per mouse, emulsified in Montanide ISA 720 adjuvant in a volume of 100 μL in the tail base. rAdPbT was administered at 1x10^8^ PFU intranasally (IN), subcutaneously (SC) in the base tail or intraperitoneally (IP). ELISA plates were coated with 0.5 μg/well of recombinant HBcAg. Pre-immune serum was used to calculate absorbance cut-off values. * *p* < 0.05 and **p < 0.01 as analyzed by Student t-test (n = 3 mice/group).
(TIF)
