# Supplementary figures and images for: Recombinant vaccines of a CD4+ T-cell epitope promote efficient control of Paracoccidioides brasiliensis burden by restraining primary organ infection
Source: PLoS Negl Trop Dis. 2017 Sep 22;11(9):e0005927. doi: 10.1371/journal.pntd.0005927 (PMC5627964; doi:10.1371/journal.pntd.0005927)

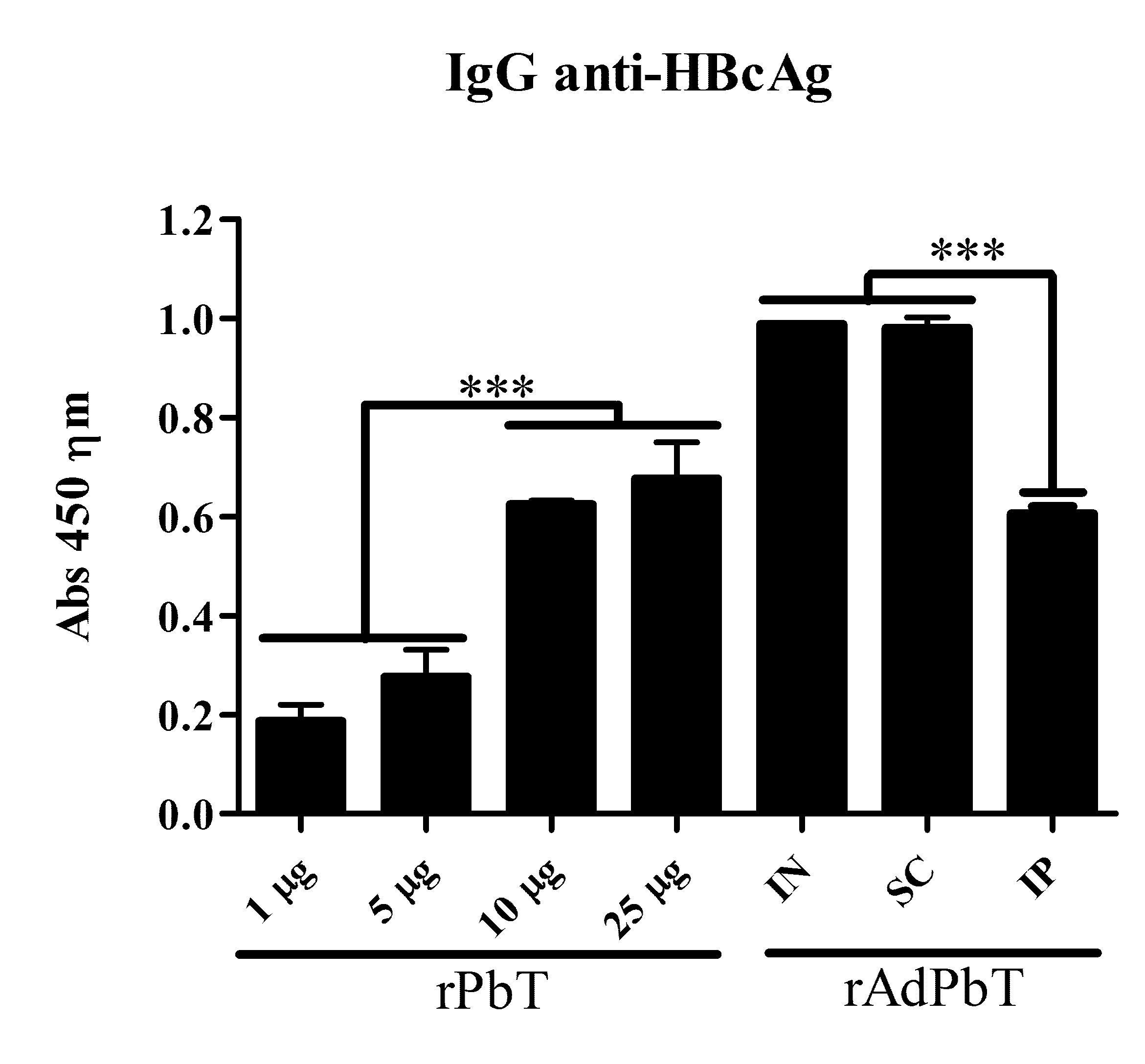

Supplement: S1 Fig — Detection of IgG anti-HBcAg in sera of mice immunized with rPbT and rAdPbT collected 15 days after immunization. rPbT was administered subcutaneously at 1, 5, 10 and 25 μg per mouse, emulsified in Montanide ISA 720 adjuvant in a volume of 100 μL in the tail base. rAdPbT was administered at 1x10^8^ PFU intranasally (IN), subcutaneously (SC) in the base tail or intraperitoneally (IP). ELISA plates were coated with 0.5 μg/well of recombinant HBcAg. Pre-immune serum was used to calculate absorbance cut-off values. * p < 0.05 and **p < 0.01 as analyzed by Student t-test (n = 3 mice/group). (TIF) [file pntd.0005927.s001.tif]
